# Identification and characterization of a moonlighting protein-enolase for surface display in *Streptococcus thermophilus*

**DOI:** 10.1186/s12934-020-01389-y

**Published:** 2020-06-17

**Authors:** Yingli Mu, Yongping Xin, Tingting Guo, Jian Kong

**Affiliations:** State Key Laboratory of Microbial Technology, Shandong University, 72 Binhai Dadao, Qingdao, 266237 People’s Republic of China

**Keywords:** Lactic acid bacteria, *Streptococcus thermophilus*, Surface display system, Enolase

## Abstract

**Background:**

*Streptococcus thermophilus* is an important food starter and receiving more attention to serve as cell factories for production of high-valued metabolites. However, the low yields of intracellular or extracellular expression of biotechnological and biomedical proteins limit its practical applications.

**Results:**

Here, an enolase EnoM was identified from *S. thermophilus* CGMCC7.179 with about 94% identities to the surface-located enolases from other *Streptococcus* spp. strains. The EnoM was used as an anchor to achieve surface display in *S. thermophilus* using GFP as a reporter. After respectively mixing the GFP-EnoM fusion protein or GFP with *S. thermophilus* cells in vitro, the relative fluorescence units (RFU) of the *S. thermophilus* cells with GFP-EnoM was 80-folds higher than that with purified GFP. The sharp decrease in the RFU of sodium dodecyl sulfate (SDS) pretreated cells compared to those of non-pretreated cells demonstrated that the membrane proteins were the binding ligand of EnoM. Furthermore, an engineered β-galactosidase (β-Gal) was also successfully displayed on the cell surface of *S. thermophilus* CGMCC7.179 and the relative activity of the immobilized β-Gal remained up to 64% after reused 8 times. Finally, we also demonstrated that EnoM could be used as an anchor for surface display in *L. casei*, *L. bulgaricus*, *L. lactis* and *Leuconostoc lactis*.

**Conclusion:**

To our knowledge, EnoM from *S. thermophilus* was firstly identified as an anchor and successfully achieved surface display in LAB. The EnoM-based surface display system provided a novel strategy for the enzyme immobilization.

## Background

Lactic acid bacteria (LAB) are widely used in dairy fermented products and pharmaceutical industry owing to their generally regarded as safe (GRAS) status [1]. Moreover, because of their ability to survive in harsh conditions including low pH, the gastro-intestinal tract (GIT) and harsh industrial fermentation processes, LAB have gained more attention to serve as cell factories for delivering bioactive molecules or vaccines to mucosal tissues [2–5] and production of high-valuable enzymes [6–9].

Recently, several studies have focused on the development of convenient and widespread genetic engineering tools for intracellular or extracellular expression of heterologous proteins in LAB [10–13]. However, the intracellular expression of proteins may confer huge burden to the host cells and result in the formation of inclusion bodies while the extracellularly expressed proteins could be degraded and exposed to harsh conditions [14–16]. In these cases, it is well accepted to display the heterologous proteins on the surface of bacterial cells. Therefore, many attempts have been made to establish surface display systems with various anchors in lactic acid bacteria, especially in *Lactobacilli* and *Lactococci* for production of heterologous proteins, such as enzymes and bioactive molecules [17–22]. As a landmark, the M6 protein from *Streptococcus pyogenes* D471 was well characterized containing LPXTG motif and used as an anchor to display the nuclease NucA on the cell surface of *L. lactis* [23]. Subsequently, several proteins have been adopted as anchors to successfully display active enzymes or bioactive molecules on the cell surface of LAB [18, 24–28]. However, the efficiency and applicability of these surface display systems are usually strain specific.

Of all the LAB, *Streptococcus thermophilus* is the second most important starter culture after *L. lactis* and was cocultured with *Lactobacillus delbrueckii* subsp. *bulgaricus* to manufacture traditional yogurt [29]. Moreover, *S. thermophilus* was able to produce a variety of health-promoting bioactive components (γ-aminobutyric acid and folate) and decrease the risk of pathologies [30, 31]. Therefore, *S. thermophilus* has attracted more attention to serve as cell factories to produce heterologous proteins [6, 32, 33]. Displaying heterologous proteins such as active enzymes and antigens on the cell surface of *S. thermophilus* could not only alleviate growth and metabolic burdens, but make target proteins well-protected, stable and reusable. Nowadays, a well-known proteinase PrtS based on the function of a sortase SrtA from *S. thermophilus* LMD-9 was employed to display heterologous proteins in *S. thermophilus* [27]. However, only a few *S. thermophilus* strains displayed this proteinase activity [34], indicating that the PrtS-based surface display system was only suitable for specific strains. Moreover, comparative genomic analysis revealed that the cell surface proteins of *S. thermophilus* were 2/3 less than that of *L. lactis* [35], which was considered as a huge obstacle for the development of surface display systems for *S. thermophilus.* Therefore, exploring anchors to effectively and stably display heterologous proteins on the cell surface of *S. thermophilus* strains for biotechnological and biomedical applications is very instant.

Enolase is a 41–50 kDa enzyme catalyzing the conversion of 2-phosphoglyceric acid (2-PGA) to phosphoenolpyruvic acid (PEP) in cytoplasm. Also, most of them were characterized as a moonlighting protein secreted to the cell surface of bacteria as well as LAB [36–39]. In this study, we demonstrated the enolase-EnoM from *S. thermophilus* could bind to the cell surface. Firstly, an enolase coding gene-*enoM* was identified in the genome of *S. thermophilus* CGMCC7.179 by sequence analysis and investigated its binding ability as an anchor to drive the GFP or β-Gal to the surface of *S. thermophilus* and other LABs in vitro. The aim of this study is to establish an EnoM-based surface display system for *S. thermophilus* as well as other LAB strains.

## Results

### Identification of enolase in *S. thermophilus* CGMCC7.179

**Fig. 1:**
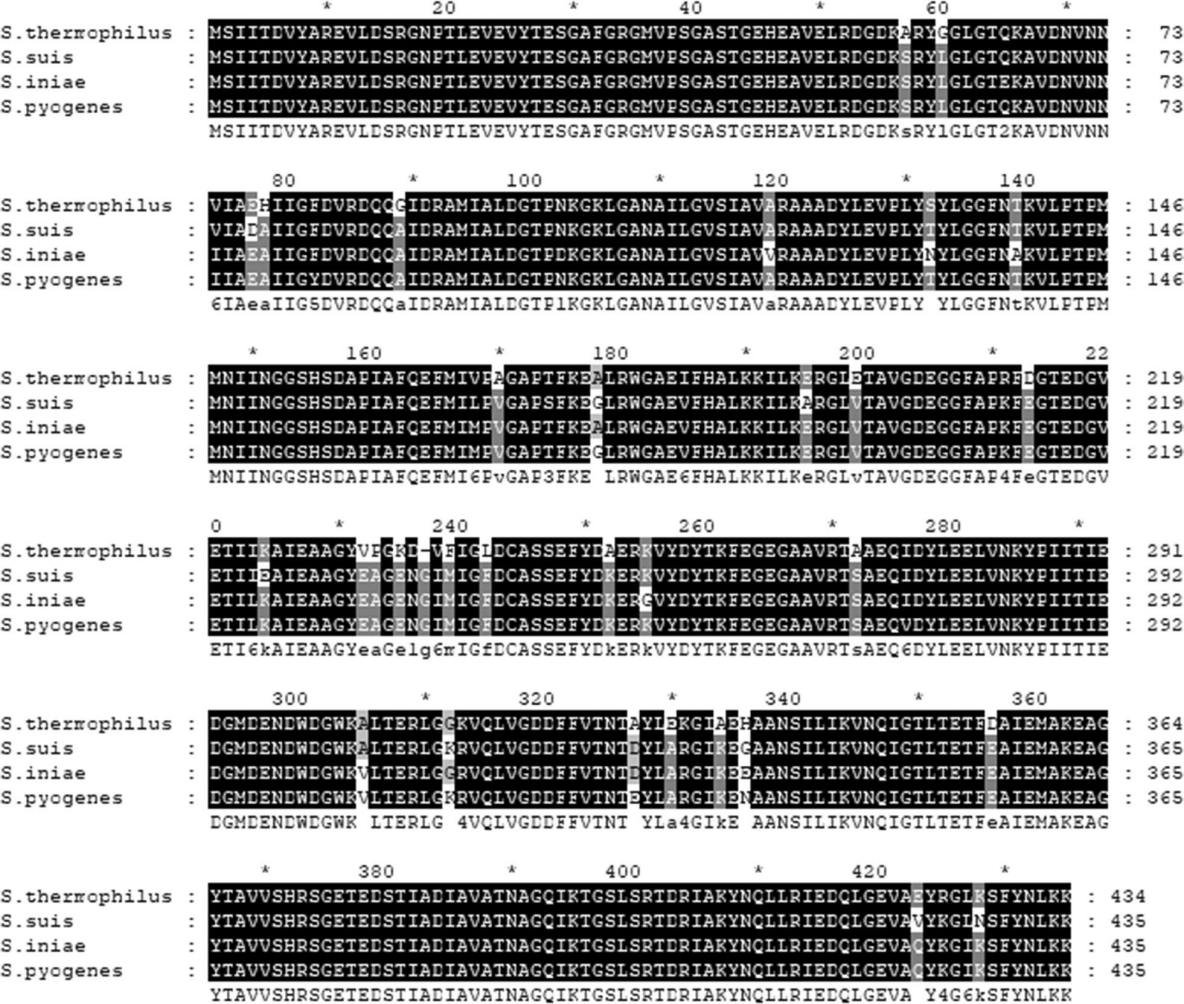
A multiple-sequence alignment of EnoM from *S. thermophilus* CGMCC7.179 to those from *S. suis* CZ130302 (Accession Number: ATZ03263), *S. iniae* DGX07 (Accession Number: AGT63054) and *S. pyogenes* Manfredo (Accession Number: CAM30572)

To characterize whether the enolase from *S. thermophilus* could bind to microbial surface, an enolase encoding gene *enoM* was identified in the genome of *S. thermophilus* CGMCC7.179. It was 1305 bp in size and composed of 435 amino acid residues. Multiple-sequence alignment results showed that the deduced EnoM protein sequence of *S. thermophilus* shared 94% similarities to the enolases of *S. suis*, *S. iniae* and *S. pyogenes* (Fig. 1), suggesting that EnoM of *S. thermophilus* CGMCC7.179 may serve as an anchor to bind to the cell surface. Moreover, functional prediction revealed that EnoM was composed of a N-terminal domain and a C-terminal domain containing 127 and 307 amino acid residues, respectively. However, no signal peptide or membrane-spanning domain was found in the protein sequence of EnoM.

### Justification of the anchoring function of EnoM from *S. thermophilus* CGMCC7.179

**Fig. 2:**
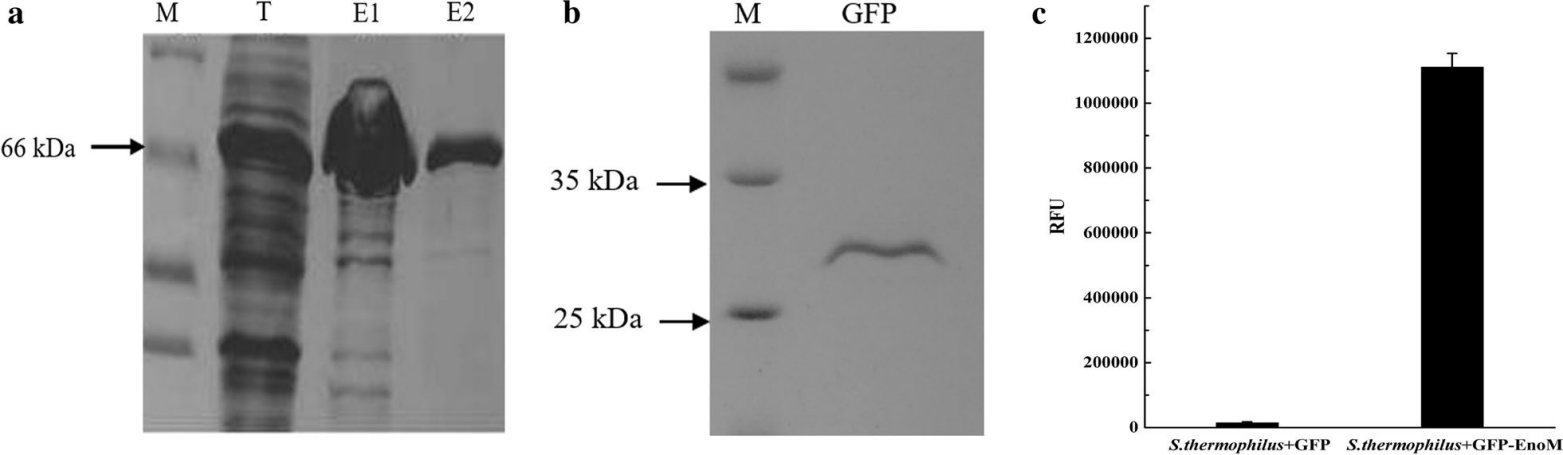
SDS-PAGE analysis of the purified GFP-EnoM fusion protein (**a**) and GFP protein (**b**) from *E. coli*. Lane M: Protein Marker; T: Total proteins in the lysate supernatant of *E. coli*/pET28a (+)-*gfp*-*enoM*; Lanes E1 and E2: two sequential eluted samples of purified GFP-EnoM. **c** The anchoring function of EnoM. Results are the averages from three independent experiments with standard deviations indicated by error bars

To investigate the function of EnoM to serve as an anchor, the successfully purified proteins GFP-EnoM (74 kDa) and GFP (26 kDa) (Fig. 2a, b) were respectively incubated with the cells of *S. thermophilus* CGMCC7.179 in vitro and the RFU of the cells were measured. As shown in Fig. 2c, the RFU of *S. thermophilus* cells mixed with GFP-EnoM fusion protein was about 1.1 × 10^5^, 80-folds higher than that of cells with GFP protein as a control, suggesting that the EnoM protein could effectively drive GFP to bind on the cell surface of *S. thermophilus*.

### Binding ability of the GFP-EnoM to the *S. thermophilus* CGMCC7.179 cells pretreated with chemical agents

**Fig. 3:**
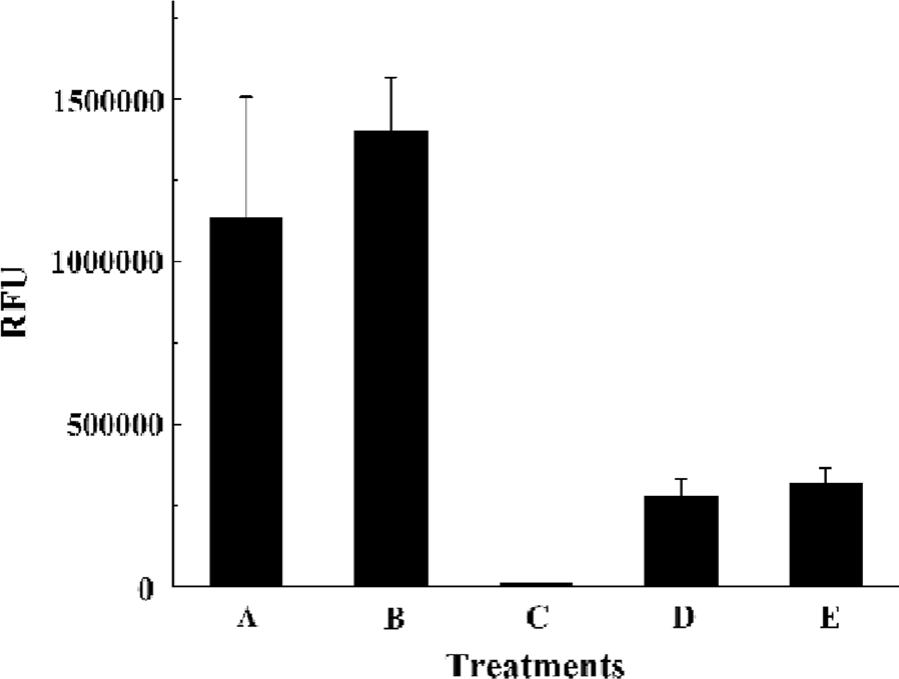
Effects of different pretreatments of *S. thermophilus* cells on the EnoM-binding capacity. Bar A, heating at 100 °C for 10 min; bar B, 5% TCA at 100 °C for 10 min; bar C, 10% SDS at 100 °C for 10 min; bar D, no treatment; bar E, 90% acetone at room temperature for 10 min. Results are the averages from three independent experiments with standard deviations indicated by error bars

The chemical components on the cell surface played key roles in the interaction of bacteria with the heterologous proteins. To identify the elements on the cell surface interacted with EnoM, the chemical agents including trichloroacetic acid (TCA), SDS and acetone were respectively adopted to remove the teichoic acids, cell-associated proteins and the cell-wall associated lipids. After pretreating the *S. thermophilus* CGMCC7.179 cells with these three chemical agents, the binding efficiency of the fusion protein GFP-EnoM to *S. thermophilus* CGMCC7.179 cells were determined. As shown in Fig. 3, the RFU of the cells pretreated with TCA was increased. No change of the RFU was observed after acetone treatment while the RFU of the cells pretreated with SDS decreased dramatically to zero, suggesting that the cell-associated protein was a binding target for EnoM.

### Optimization of the binding efficiency of GFP-EnoM to *S. thermophilus* cells

**Fig. 4:**
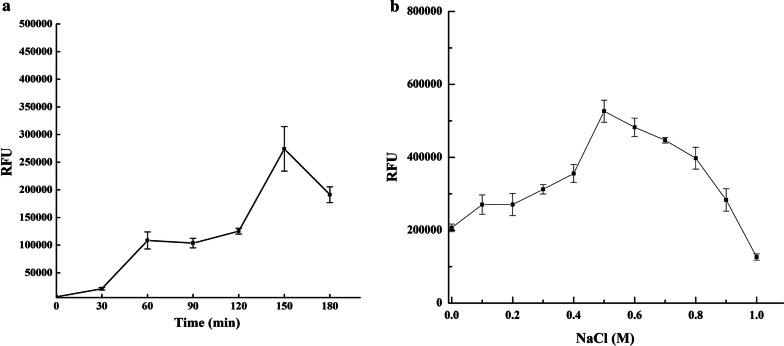
Optimization of the binding efficiency of EnoM. **a** The influence of binding time on the binding efficiency of EnoM. **b** The influence of Na^+^ on the binding efficiency of EnoM. Results are the averages from three independent experiments with standard deviations indicated by error bars

To enhance the binding efficiency of GFP-EnoM, the influence of binding time and concentration of Na^+^ were tested. As shown in Fig. 4a, the RFU of cells increased along with the binding time and reached the highest value of RFU when the cells were incubated with GFP-EnoM for 150 min. Na^+^ could enhance the binding capacity, and the optimal concentration was 0.5 M (Fig. 4b).

### Displaying β-Gal on the surface of *S. thermophilus* cells

**Fig. 5:**
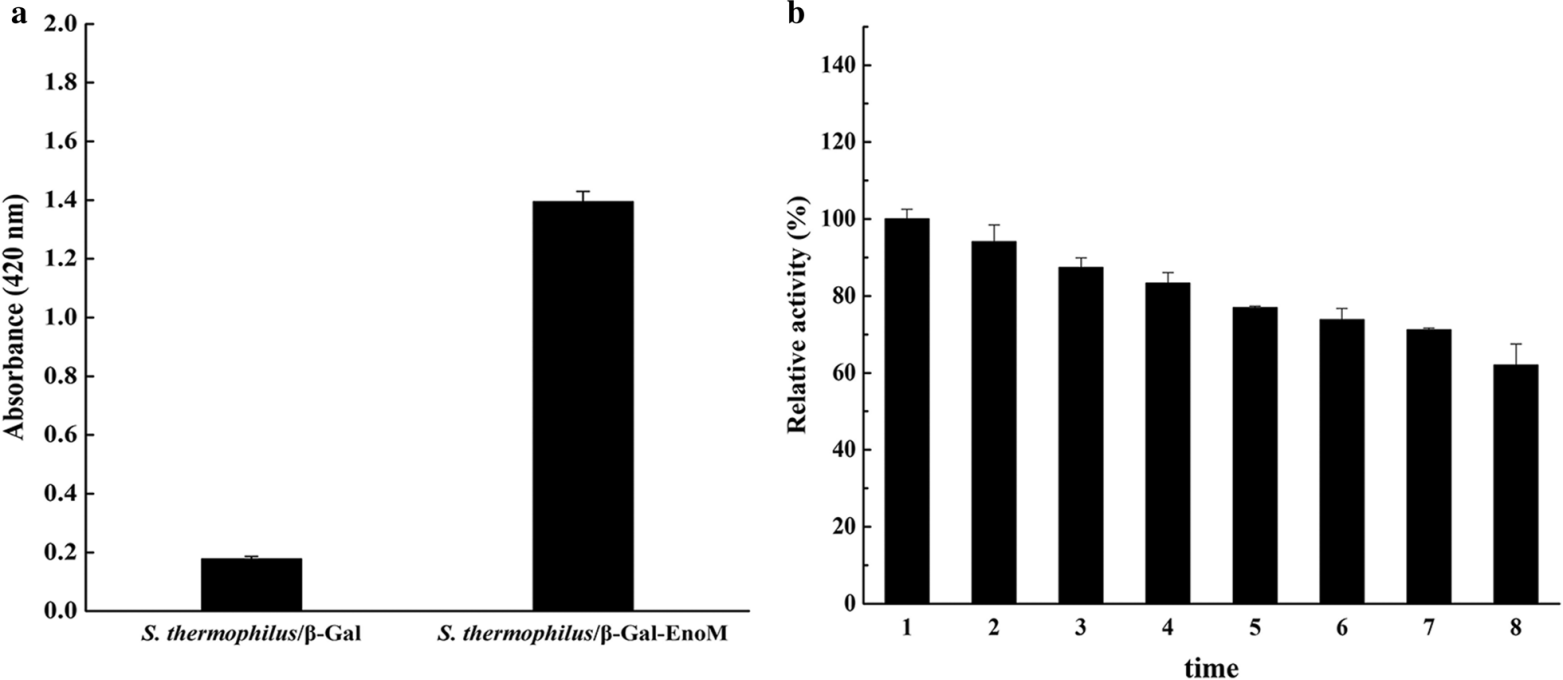
Display of β-Gal on the surface of *S. thermophilus* CGMCC7.179 cells. **a** The β-Gal activity (indicated by absorbance, 420 nm) of the *S. thermophilus* CGMCC7.179 cells bound with β-Gal-EnoM and β-Gal. **b** The stability and activity of the immobilized β-Gal. 1-8 indicated that the β-Gal bound *S. thermophilus* CGMCC7.179 cells were reused 1-8 times to react with *o*-nitrophenyl-β-d-galactoside (*o*NPG). Results are the averages from three independent experiments with standard deviations indicated by error bars

To further confirm the feasibility of the surface display system, the remodeled β-galactosidase (β-Gal) from *S. thermophilus* SDMCC050237 and the fusion protein β-Gal-EnoM were successfully expressed (data not shown). Subsequently, the whole cell extracts containing β-Gal and β-Gal-EnoM were mixed with *S. thermophilus* CGMCC7.179 cells. The β-Gal activity of the cells incubated with β-Gal-EnoM was higher than that of the cells incubated with β-Gal (Fig. 5a), indicating that β-Gal was successfully displayed on the cell surface of *S. thermophilus* CGMCC7.179 driven by the enolase EnoM. To investigate the stability and activity of the immobilized β-Gal, we reused the bound *S. thermophilus* CGMCC7.179 cells to react with *o*-nitrophenyl-β-d-galactoside (*o*NPG). Results showed that the immobilized enzyme on the cell surface could retain 64% of the maximal activity after reutilization of 8 times (Fig. 5b).

### Binding ability of EnoM to other LAB strains

**Fig. 6:**
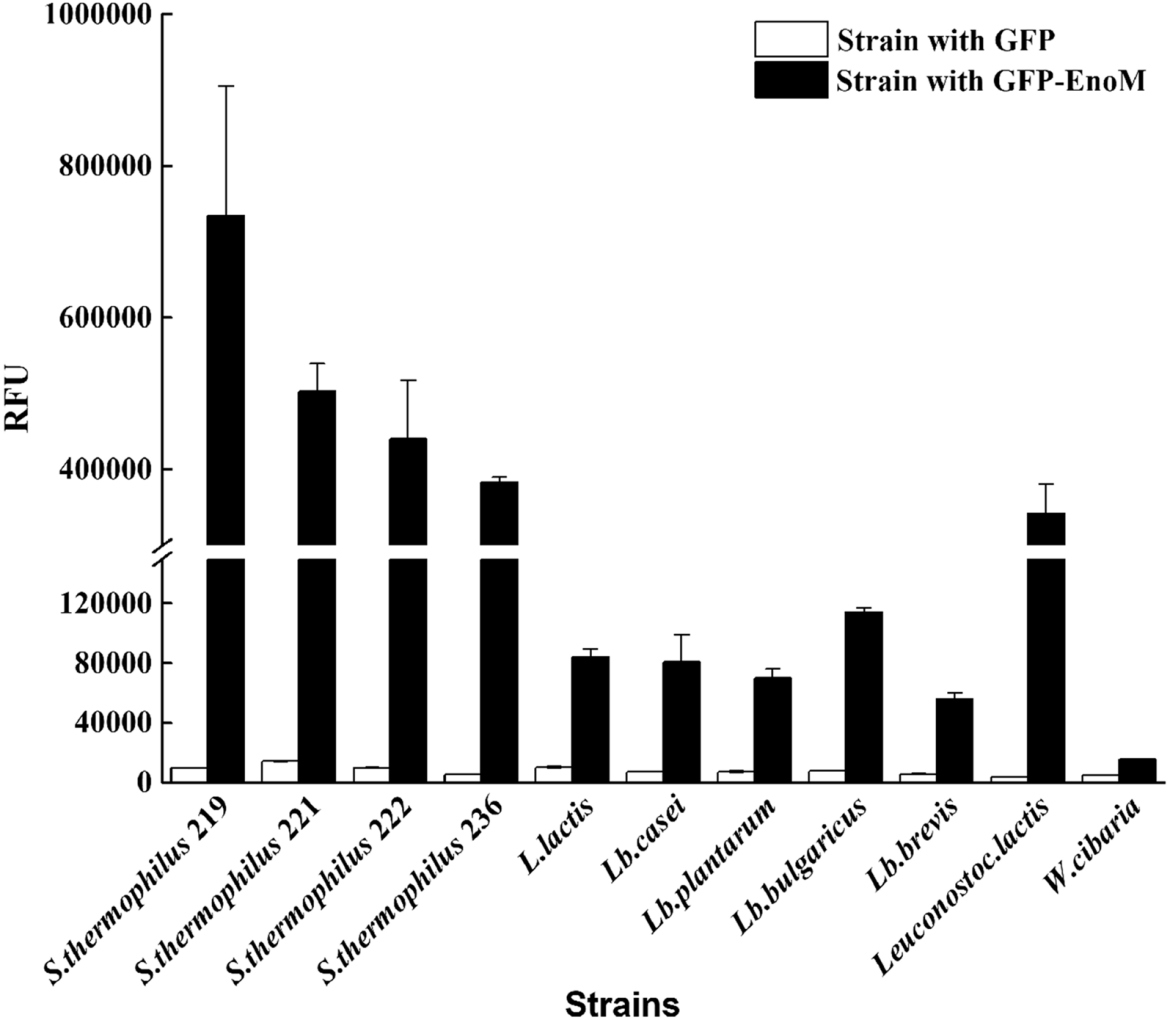
Extending the binding ability of EnoM to other LAB strains. RFU indicated that the relative fluorescence intensities of various strains after react with GFP or GFP-EnoM. Results are the averages from three independent experiments with standard deviations indicated by error bars

To explore whether EnoM could be used as an anchor to drive protein to other LAB strains, we incubated *L. plantarum* LA, *L. lactis* NZ9000, *L. delbrueckii* JB, *L. casei* SDMCC050427, *L. brevis* ATCC367, *Leuconostoc lactis* SDMCC050430, *Weissella cibaria* SDMCC050356 and several other *S. thermophilus* cells with GFP-EnoM fusion protein in vitro. As shown in Fig. 6, most LAB cells appeared higher RFU than those incubated with GFP as control. And the cells of *S. thermophilus* strains had higher binding ability with GFP-EnoM than the other LAB strains tested, indicating that the enolase EnoM as anchor showed the strain specificity. It was worth noting that the cells of *Leuconostoc lactis* SDMCC050430 showed the highest RFU among the LAB strains while *W. cibaria* SDMCC050356 displayed the lowest RFU. All the results demonstrated that EnoM could be used to display proteins on the surface of LAB strains and the binding efficiency varied with strains.

## Discussion

Recently, lactic acid bacteria have received increasing attention as cell factories for heterologous proteins production because of its GRAS status and ability to survive in the harsh environments. Also, it is widely accepted that proteins attached to the bacterial surface is better protected than a free protein [1]. However, the surface display system for *S. thermophilus* is rarely studied because there is little surface protein in *S. thermophilus* and the gene editing efficiency is quite low for most of *S. thermophilus* strains [35], limiting its application for food and pharmaceutical industry.

In this study, an α-Enolase-EnoM was identified from *S. thermophilus* CGMCC7.179 and proved to have the ability to anchor heterologous proteins to the cell surface of *S. thermophilus*, raising the possibility of developing effective and stable surface display system for *S. thermophilus*. To our knowledge, this is the first report on describing the use of α-Enolase from *S. thermophilus* as an anchor for surface display in LAB.

Recently, enolases from other Streptococci strains have been reported to be secreted and attached to the cell surface [36, 40, 41]. As like these enolases, the protein sequence of EnoM do not contain classical motifs such as signal peptidase cleavage site, membrane spanning domains or anchoring motif [37]. In this work, to explore the binding mechanism of EnoM, we pretreated the cells of *S. thermophilus* CGMCC7.179 with different chemical reagents including SDS, TCA and acetone to remove proteins, teichoic acid and lipids from cell surface respectively. The RFU of the SDS-pretreated cells decreased rapidly to zero, demonstrating that the EnoM could bind with the surface-associated proteins of *S. thermophilus* CGMCC7.179. Besides, the anchoring efficiency of EnoM was slightly enhanced by TCA treatment, suggesting that EnoM could not bind to teichoic acid. This phenomenon was different from the enolase from *L. crispatus* ST1 which bound to the lipoteichoic acid under appropriate pH value [42]. Possibly TCA treatment allowed the exposure of more binding sites for EnoM. Additionally, acetone treatment had no significant influence on the anchoring of EnoM, indicating that lipid was not the binding component of EnoM.

Moreover, the RFU of cells reached the highest level at 150 min. After that, the RFU of cells decreased significantly. This could be explained by that the binding sites were gradually saturated overtime and excessive binding time may lead to the separation between EnoM and the surface-associated proteins. Also, the RFU of bound cells was significantly influenced by the concentration of NaCl and the optimum concentration was 0.5 M, revealing that Na^+^ may affect the interaction between proteins and subsequently influence the anchoring efficiency of EnoM.

Purified enzymes are often non-reusable and suffer from the environment pressure in the industrial production and storage [18, 43–45]. In this study, the EnoM could drive the β-galactosidase on the surface of *S. thermophilus* cells in vitro, achieving the reutilization of this enzyme. Therefore, the surface display system developed in this study can provide a novel strategy for other researchers who work in the enzyme immobilization area.

Several surface display systems have been employed to fulfill the heterologous protein expression in LAB, including *S. thermophilus* [19, 20, 22, 25, 27]. Unlike the efficiency and applicability of the above systems which were usually strain specific, the EnoM mediated surface display system could be functional in most LAB strains, indicating its universality. Importantly, EnoM could effectively anchor GFP to the cell surface of *L. delbrueckii* spp. *bulgaricus* JB, in which no efficiency in vivo protein expression system has been reported, providing the possibility of using *Lb. bulgaricus* as a vehicle for delivery of bioactive molecules.

## Conclusions

An enolase-EnoM from *S. thermophilus* CGMCC7.179 was firstly described as an anchor to display heterologous proteins on the surface of different LAB strains by combining with surface-associated proteins. It provided a new strategy for the delivery of vaccines, the enzyme immobilization, the development of protective antigens in LAB.

## Materials and methods

### Plasmids, bacterial strains, and growth conditions

The strains and plasmids used in this work are listed in Table 1. *S. thermophilus* CGMCC7.179 was isolated from a traditional yogurt of Inner Mongolia, China. *S. thermophilus* strains were cultured in M17 broth (OXOID) supplemented with 1% lactose statistically at 42 °C. *Lactobacillus* spp. strains were grown in MRS broth (OXOID) statistically at 37 °C. *Lactococcus lactis* NZ9000 and *Leuconostoc lactis* SDMCC050430 were cultured in M17 broth (OXOID) containing 0.5% glucose statically at 30 °C and 37 °C, respectively. *E. coli* strains were used for standard cloning and expressing procedures and grown aerobically in Luria–Bertani (LB) broth at 37 ℃. Kanamycin and chloramphenicol were added at the final concentration of 30 µg/mL and 10 µg/mL respectively for *E. coli.*

**Table 1:** Strains and plasmids used in this study

Strain or plasmid	Genotype or characteristics	Reference or source
Strains		
Lactic acid bacteria strains		
*S. thermophilus*	Wild type	Our lab
*L. lactis* NZ9000	*L. lactis* MG1363 pepN::nisRK; commonly used host for NICE system	[48]
*L. casei* SDMCC050427	Wild type isolated from Chinese artisanal yogurt	Our lab
*L. plantarum* LA	Wild type isolated from Chinese artisanal yogurt	Our lab
*L. brevis* ATCC 367	Wild type	[49]
*L. bulgaricus* JB	Wild type isolated from Chinese commercial yogurt	Our lab
*Leuconostoc lactis* SDMCC050430	Wild type isolated from Chinese artisanal yogurt	Our lab
*W.cibaria* SDMCC050356	Wild type isolated animal feed additive	Our lab
*E. coli* strains		
DH5α	*supE44 ΔlacU169 Φ*80*lacZΔ*M15 *hsdR17 recA1 endA1 gyrA96 thi*-*1 relA1*	Novagen
BL21 (DE3)	F^−^*ompT hsdS*B (rB^−^ mB^−^) *gal dcm*	Novagen
Plasmids		
pD2*gfp*	pSec:leiss:Nuc derivative, expresses *gfp* under PD2 control, Cm^R^	[50]
pET28a (+)	Kan^r^; *E. coli* expression vector	Novagen
pET28a (+)-*gfp*	Kan^r^; pET28a (+) carrying NdeI/HindIII-digested product expressing His_6_-tagged GFP	This study
pET28a (+)-*gfp*-*enoM*	Kan^r^; pET28a (+) carrying NdeI/HindIII-digested product expressing His_6_-tagged GFP-EnoM	This study
pET28a (+)-*gal*	Kan^r^; pET28a (+) carrying NcoI/XhoI-digested product expressing His_6_-tagged engineered β-Gal from *S. thermophilus* SDMCC050237	Unpublished data
pETDuet1	Ap^r^; *E. coli* expression vector	This study
pETDuet1-*gal*-*enoM*	Ap^r^; pETDuet1 carrying BamHI/SalI-digested product expressing His_6_-tagged β-Gal-enoM	This study

### Bioinformatic analysis

The whole genome sequence of *S. thermophilus* CGMCC7.179 was analyzed and gene functional prediction was performed using the software Glimmer 3.0. Multiple-sequence alignment was performed using GeneDoc and Mega 7. The amino acid sequence of EnoM was aligned and analyzed with those of the enolases from *Streptococcus suis*, *Streptococcus iniae* and *Streptococcus pyogenes* obtained from NCBI website (https://www.ncbi.nlm.nih.gov/). Also, the functional prediction of EnoM was performed by Interpro website (http://www.ebi.ac.uk/interpro/).

### Construction of plasmids

The primers used in this study are listed in Table 2. The *gfp* gene was cloned with primer pair gfpF/gfpR1 using plasmid pD2*gfp* as a template. The resulted fragment was digested with NdeI and HindIII and ligated into the corresponding sites of pET28a (+) to construct plasmid pET28a (+)-*gfp*. Besides, the *gfp* gene amplified with primer pair gfpF/gfpR2 using pD2*gfp* as a template was fused with the *enoM* gene cloned with primer pair gfp-enoF/enoR1 from the genome of *S. thermophilus* CGMCC7.179 by an overlapping PCR. The fused *gfp*-*enoM* fragment was digested with NdeI and HindIII and inserted into the same sites of pET28a (+), resulting the plasmid pET28a (+)-*gfp*-*enoM*.

**Table 2:** Oligonucleotide primers used in this study

Primer	Sequence (5ʹ–3ʹ)	Restriction site(s)
gfpF	GGAATTCCATATGAGCAAAGGAGAAGAACTTTTCA	NdeI
gfpR1	CCCAAGCTTGTAGAGCTCATCCATGCCATGTGTA	HindIII
gfpR2	GTAGAGCTCATCCATGCCATGTGTA	
gfp-enoF	ATGGCATGGATGAGCTCTACATGTCAATTATTACTGATGTCTA	
enoR1	CCCAAGCTTTTATTTTTTCAAGTTGTAGAATGAT	HindIII
galF1	CATGCCATGAACATGACTGAAAAAATTCAAA	NcoI
galR2	CCGCTCGAGATTTAGTGGTTCAATCATGAAGCTT	XhoI
galF2	CGGGATCCAATGAACATGACTGAAAAAATTCAAA	BamHI
galR2	ATTTAGTGGTTCAATCATGAAGCTT	
gal-enoF	TCATGATTGAACCACTAAATATGTCAATTATTACTGATGT	
enoR2	ACGCGTCGACTTATTTTTTCAAGTTGTAGAATGAT	SalI

The *β*-*gal* amplified with primer pair galF2/galR2 from the plasmid pET28a (+)-*gal* (unpublished data) was fused with the *enoM* gene cloned with primer pair gal-enoF/enoR2 from the genome of *S. thermophilus* CGMCC7.179 by an overlapping PCR. The fused *gal*-*enoM* fragment was digested with BamHI and SalI and ligased to the corresponding sites of pETDuet1 to obtain the plasmid pETDuet1-*gal*-*enoM*.

### Overexpression and purification of recombinant proteins in *E. coli*

*E. coli* strains harboring the recombinant plasmids were cultured overnight at 37 ℃ in LB media containing 30 µg/mL kanamycin. Subsequently, the overnight cultures were diluted 50-folds in 100 mL fresh LB broth containing 30 µg/mL kanamycin and the expression of recombinant proteins was induced with 0.1 mM isopropyl-β-d-thiogalactopyranoside (IPTG) when the OD_600_ of the cultures reached 0.8. After incubated at 16 ℃ for 18 h, target proteins were purified following the steps described previously [46]. Briefly, cells were harvested by centrifugation, washed with phosphate-buffered saline (PBS: 137 mM NaCl, 2.7 mM KCl, 10 mM Na_2_HPO_4_, 2 mM KH_2_PO_4_, pH 7.4). The cells were resuspended in 8 mL binding buffer (20 mM sodium phosphate, 20 mM imidazole, 500 mM NaCl, pH 7.4) and ultrasonicated on ice at 400 wt for two cycles (one cycle consists of 99 periods of ultrasonication for 5 s with intermission of 5 s). The supernatant obtained by centrifugation at 10,000 g for 20 min was passed through a filter (0.22-µm pore size; Millipore) and applied to HisTrap FF crude columns (GE healthcare) balanced with binding buffer. Subsequently, the columns were washed with washing buffer (20 mM sodium phosphate, 40 mM imidazole, 500 mM NaCl, pH 7.4) and the His-tagged proteins were eluted with elution buffer (20 mM sodium phosphate, 500 mM imidazole, 500 mM NaCl, pH 7.4). The concentration of purified proteins was determined using a BCA protein assay kit (Sangon Biotech, China) with bovine serum albumin as the standard protein. The eluted samples were analyzed by SDS-PAGE.

### Exposure of the heterologous proteins on the surface of *S. thermophilus* CGMCC7.179

The binding reaction was performed as previously described [25]. Briefly, *S. thermophilus* CGMCC7.179 was cultured overnight and collected by centrifugation. After being washed three times with PBS, the value of OD_600_ was adjusted to 1.0 using PBS buffer. Then the cells were incubated with equal volume of the eluted samples containing adequate GFP, GFP-EnoM, β-Gal or β-Gal-EnoM proteins for 1 h or 2.5 h at 42 °C, 120 rpm respectively. After reaction, the cells were collected by centrifugation at 12, 000 g for 5 min and washed five times with 1 mL PBS. The cells were resuspended in PBS with vortex mixing and the binding efficiency of the GFP and β-Gal were determined by whole-cell fluorescence measurement and analysis of β-Gal enzyme activity, respectively.

### Fluorescence assay

Fluorescence intensity was determined according to our previous work [47]. For each sample, three repetitions were performed.

### Measurement of the relative enzyme activity of β-Gal

The enzyme activity of β-Gal was assayed using *o*NPG as substrate for 10 min and the absorbance was measured under 420 nm. To determinate the stability of the immobilized enzyme, we mixed the cells with β-Gal-EnoM and collected the supernatant to detected A_420_. The cells were collected by centrifugation, washed with PBS and resuspended in PBS and reused “n” times for detection. For each sample, three repetitions were performed. The relative enzyme activity was designed as: (The A_420_ value of the β-Gal after reused “n” times/The A_420_ value of the initial β-Gal) × 100%.

### The binding ability of GFP-EnoM after treatments with chemical reagents

The *S. thermophilus* cells were collected, washed and resuspended in PBS as mentioned above. Then the value of OD_600_ of cells was adjusted to 1.0 before being treated with different chemical reagents including 5% TCA, 10% SDS at 100 °C for 10 min and acetone at room temperature for 10 min. After treatments, the cells were washed with PBS to remove the left chemicals and resuspended in PBS. The binding experiments were performed and RFU was determined as described above. For each sample, three repetitions were performed.

### Extending the EnoM-based surface display system to other LAB strains

LAB strains were cultured overnight and binding experiments were performed as described above. Briefly, 1 mL culture was collected, washed three times and resuspended in 1 mL PBS. Then the cells were incubated with abundant GFP and GFP-EnoM proteins respectively. Consequently, the cells were collected by centrifugation, washed and resuspended in 1 mL PBS. The RFU of the cells bound with GFP and GFP-EnoM were calculated. For each sample, three repetitions were performed.

## Data Availability

The data sets supporting the conclusions of this article are included within the article.

## Abbreviations

LAB: Lactic acid bacteria
GRAS: Generally regarded as safe
RFU: Relative fluorescence units
GFP: Green fluorescence protein
